# Mechanism of injury and disposition of severe polytrauma patients at Kalafong Hospital 2018/2019

**DOI:** 10.4102/jcmsa.v2i1.79

**Published:** 2024-08-13

**Authors:** Onke P. Tuswa, Maria M. Geyser, Vidya Lalloo, Almien Smit

**Affiliations:** 1 Division of Emergency Medicine, Faculty of Health Science, University of Pretoria, Pretoria, South Africa; 2 Division of Emergency Medicine, Faculty of Health Science, Tembisa Provincial Tertiary Hospital, Johannesburg, South Africa; 3 Division of Emergency, Faculty of Health Science, Kalafong Provincial Tertiary Hospital, Pretoria, South Africa; 4 Division of Emergency Medicine, Faculty of Health Science, Kalafong Provincial Tertiary Hospital, Pretoria, South Africa

**Keywords:** polytrauma, severe polytrauma, disposition, mechanism of injury, emergency department length of stay, emergency department, injury severity scores, low- to middle-income countries

## Abstract

**Background:**

The most common mechanisms of injury (MOI) for trauma patients in South Africa (SA) are transport-related and interpersonal violence. South Africa is rated among the highest in the world with regard to mortality from injuries sustained, yet many tertiary public hospitals do not have dedicated trauma teams. The aim of this study was to observe the MOI and disposition of severe polytrauma patients presenting to Kalafong Provincial Tertiary Hospital (KPTH) emergency department (ED). A secondary aim was to observe associations between the MOI, severity of injuries, and patient outcome.

**Methods:**

Retrospective descriptive data were collected on all severe polytrauma patients who presented at KPTH ED from 01 July 2018 to 31 December 2019.

**Results:**

Most severe polytrauma patients were males (62%). Motor vehicle accidents (MVAs) were the most prevalent mechanism, which accounted for 28% of MOI. Patients involved in pedestrian vehicle accidents (PVAs) were most severely injured. Most patients were admitted to the intensive care unit (ICU) (36%) or high care unit (HCU) (26%). The waiting time for ICU admissions ranged between 6.4 and 7.7 h. Fourteen per cent of patients demised in the ED before admission.

**Conclusion:**

While MVA was the predominant MOI for severe polytrauma patients presenting to KPTH ED, patients involved in PVAs were most severely injured. The majority of severe polytrauma patients were admitted to ICU.

**Contribution:**

This study contributes to the limited literature on the important topic of severe polytrauma in Gauteng, SA. It may influence staffing decisions and motivate for province-wide trauma database collection.

## Background

Worldwide and across genders, trauma has been found to be the sixth leading cause of death in persons younger than 40 years of age.^1^ In South Africa (SA), both interpersonal violence and transport-related trauma are identified as leading causes of trauma.^2^

Trauma is defined as an injury that can be intentional or unintentional.^2^ Polytrauma, as per the Berlin Definition^3^ in 2014, is defined as ≥ 2 injuries that are ≥ 3 on the Abbreviated Injury Scale (AIS) and have 1 or more additional variable(s) from five physiologic parameters.

These parameters include:

1. Level of consciousness – Glasgow Coma Scale (GCS) ≤ 8
2. Hypotension – systolic blood pressure ≤ 90 mmHg
3. Acidosis – base deficit ≥ 6.0
4. Coagulopathy – international normalised ratio ≥ 1.4 or partial thromboplastin time ≥ 40 s
5. Age ≥ 70 years old

In polytrauma patients, time to definitive care is a critical factor influencing patients’ survival.^4^ Definitive care might entail taking a patient to theatre for damage control surgery, fixation of fractures or admission to intensive care unit (ICU) or high care unit (HCU) for continued resuscitation and monitoring. Managing severe polytrauma patients requires dedicated time by multidisciplinary teams. When inadequately managed, injuries are missed, leading to complications with increased hospital length of stay (LOS) and mortality.^1^

Literature shows that of trauma patients who die in hospital, 55% die within 24 h of arrival.^4^ These patients have a higher injury severity score (ISS) and greater blood transfusion requirements.^4^

Trauma in South African context: South Africa has the highest trauma-associated mortality rate in the world.^2^ We also boast injury rates that are six times the global rate, and road traffic injuries in particular, are double the global rate.^4^ Unique to SA is that pedestrians account for more than 50% of all the road traffic fatalities.^1^ Interpersonal violence is a major cause of trauma in the Eastern Cape, KwaZulu-Natal and Western Cape.^5^ Interpersonal violence injury is defined as those resulting from violence of a physical or sexual nature including that between family members and intimates, and between acquaintances and strangers.^5^

Hardcastle et al.^3^ mention that injuries in low- and middle-income countries (LMICs) are largely because of violence or are transport-related and occur mostly among young healthy men of low socio-economic status, influenced by alcohol and drug misuse.

Most South African public hospitals are faced with overcrowding, understaffing and insufficient resources.^2^ The system is challenged further when an acutely injured severe polytrauma patient arrives in the emergency department (ED). A dedicated trauma team is ideal to adequately resuscitate the patient and manage complex injuries.^3^

### Injury severity scores

It is important for clinicians to estimate the severity of injuries to assist in prognostication and disposition decisions. Most injury severity scores capture the physical and cognitive condition of polytrauma patients.^3^ Utilising scoring systems may increase the quality of management of trauma patients in the following ways^6^:

- Objectively determine the severity of injuries that patients have sustained. This will guide decisions about the appropriate level of health institution the patients should be transported to.
- Assist in identifying patients who need to be prioritised.
- The physiological parameters associated with increased early mortality will be identified and used to risk stratify patients.
- Creation of an epidemiological database regarding injuries incurred and their severity.

The scoring system used in this study is the ISS. The ISS was developed in 1974 by Baker et al. of Johns Hopkins Hospital.^7^ The ISS is calculated as the sum of the squares of the highest Abbreviated Injury Scale (AIS) in each of the three most severely injured anatomical areas.^7^ If a patient has an AIS of six in any body system, they are automatically assigned an ISS of 75^7^ (see Appendix 1 for description)^8^.

Abbreviated Injury Scale is an anatomically based injury scoring system that classifies each injury by body region on a six-point scale. The degree of each lesion is measured from 1 – being minor injury to a maximum of 6 – being un-survivable injuries^3^ (see Appendix 2)^8^.

Kalafong Provincial Tertiary Hospital (KPTH) is a tertiary hospital in Gauteng Province, SA. The surgical department has a specific firm consisting of general surgeons who manage trauma patients during the day; however, they do not have 24-h trauma surgery cover, nor do they have a qualified trauma surgeon on site. Hence, the care of trauma patients falls to the general surgeon on call. This is less than ideal as the general surgeon maybe overwhelmed and may not be equipped to manage both traumatic and non-traumatic surgical emergencies at once.

Noting the mechanisms of injury (MOI), ISS and disposition of severe polytrauma patients presenting to KPTH ED will provide a clearer picture of the burden of trauma seen at KPTH. It is the hope of the authors that this picture will inform management decisions such as resource allocation to the ED, surgical departments, ICU and HC units.

The aim of this study was to observe the mechanism of injury and the disposition of severe polytrauma patients, presenting to the ED of KPTH from 01 July 2018 to 31 December 2019.

Our objectives included the following:

- To determine whether an association exists between the mechanism of injury and the severity of injuries sustained using the ISS.
- To determine an association between mechanism of injury and the initial disposition of patients from the ED (i.e., theatre, ICU, HCU and general ward).
- To note the time taken for disposition of patients in the ED.
- To inform KPTH management about the findings of this study, which may facilitate informed resource allocation to the relevant departments.

## Methods

The study design was a retrospective audit of the files of patients who presented with severe polytrauma at KPTH ED. Kalafong Provincial Tertiary Hospital is a public hospital, situated on the outskirts of the western part of Pretoria. It is affiliated to the University of Pretoria and is governed by the Tshwane Municipality, Gauteng province, SA.

Permission to access the files of patients was granted from the CEO of the hospital. Data were collected retrospectively using the files of polytrauma patients identified in the resuscitation unit register. A total of 208 patients were identified in the register at the KPTH ED. At the filing department in KPTH, a minimum of 30 files were requested per day. Data were extracted on-site and the files were returned on the same day.

The files of identified 208 polytrauma patients were reviewed. Data were collected, including demographics, information needed to calculate the ISS, MOI, LOS, disposition and whether the patient demised in the ED or not. The ISS was calculated on each of these patients. Those patients with an ISS of 16 and above were classified as severe polytrauma and included in the study. To ensure the quality of data extraction, a data sheet was used.

The ISS is an anatomical score that enumerates multiple injuries sustained.^7^ All injuries are scored according to severity (1 = mild injury to 6 = fatal injury). Injuries are assessed with the AIS. Overall, the ISS consists of the squared scores of the three most severely affected AIS regions. The following 6 of the 9 AIS regions are included in the ISS: head and neck, face, chest, abdomen, pelvis, extremities and external (soft tissue). If a patient has an AIS of 6 in any body system, they are automatically assigned an ISS of 75 (see Appendix 1).

The data sheet comprised of the following:

- Demographics of the patient
- Information to confirm that the patient meets the criteria of a polytrauma patient (Berlin definition): initial vital signs of the patient taken by EMS at the scene, initial vitals of the patient taken in the ED and initial ABG and/or VBG parameters.
- Information necessary to calculate the ISS to define severe polytrauma.
- Details of the injury sustained, including the different systems involved and severity.
- Mechanism of injury:
  ■ Motor vehicle accident (MVA) (including if patients were ejected or not)
  ■ Pedestrian vehicle accident (PVA)
  ■ Motorbike accident (BMA)
  ■ Fall from height
  ■ Submersion injuries
  ■ Burns
  ■ Interpersonal violence (Mob assault, gunshot wound [GSW]).

Interpersonal violence was originally stipulated on the data sheet; however, because only two categories of interpersonal violence were observed during data collection, it was changed to mob assault and GSW to allow better clarity on the MOI:

- Initial disposition of patients from the ED to a general ward, HC, ICU, theatre, or transfer to another hospital. Patient demise in the ED was included in this group.
- Duration of waiting time to disposition from the ED.

The population sample consisted of all patients who presented to KPTH ED with severe polytrauma over an 18-month period, from 01 July 2018 to 31 December 2019.

### Inclusion criteria

- Severe polytrauma patients presenting to KPTH ED, who met the criteria for severity according to an ISS ≥ 16
- Age 18 years and older

### Exclusion criteria

- Patients who did not meet the criteria for severe polytrauma; ISS < 16.
- Patients who were younger than 18 years of age.

### Data analysis

Descriptive statistics including mean, median, standard deviation and interquartile range (IQR) were used to describe continuous variables. Frequencies and proportions were used to describe the categorical variables. The chi-square test was used to test for associations between categorical variables such as mechanism of injury and disposition. One-way analysis of variance (ANOVA) or a non-parametric alternative, was used to compare the mean ISS scores of the mechanism groups. Pearson’s correlations were calculated for pairs of continuous variables. Tests were evaluated at a 5% level of significance. All analysis was performed using STATA 15.

### Ethical considerations

The study was a retrospective audit with no interventions undertaken by the investigators. Identifying patients’ details were not included on the password-protected data capture sheet. Consent was obtained from the chief executive office of the hospital and the Clinical Head of the Emergency Department at KPTH. Approval was granted by the Faculty of Health Sciences Research Ethics Committee, University of Pretoria (Ethics approval number: 477/2020).

## Results

In all, 208 files were identified as polytrauma from the ED register and requested from records at KPTH. Only 191 files were included in the study. All missing files were accounted for. Five files were untraceable by the records department. Four files were excluded as the injuries did not meet the criteria for severe polytrauma as per ISS and eight files were excluded under submersion injury because the patients were less than 18 years of age.

Black males between the ages of 26 years and 35 years were most involved in severe polytrauma. The most common MOI was MVAs (28%), although the most severely injured patients were involved in PVAs (mean: ISS = 45). The most common disposition was to ICU (36%). Patients being transferred to ICU had the longest waiting times in the ED (mean: 7.7 h). The overall mortality of severe polytrauma patients in the ED was 14% with the highest mortality rate (5%) in patients who were involved in a mob assault. Most of these patients sustained severe head injuries.

Table 1 demonstrates that the majority (62%) of the patients who presented at KPTH with severe polytrauma (ISS ≥ 16) were males. Females comprised 38% of the total patients. Eighty-one per cent of patients were black people, 11% were white people, 8% were Indian people and 0% were mixed race people. Most patients were in the age group 26 years to 35 years (36%), followed by the age groups 18 years to 25 years (21%) and 36 years to 45 years (20%). Patients older than 65 years of age presented the least, at 3%.

**TABLE 1:** Demographics of severe polytrauma patients at Kalafong Provincial Tertiary Hospital emergency department.

Demographics	Frequency	Percentage (%)
**Gender**		
Female	72	38
Male	119	62
Total	191	100
**Ethnic group**		
Black people	154	81
Mixed race people	0	0
Indian people	16	8
White people	21	11
Total	191	100
**Age group (years)**		
18–25	41	22
26–35	69	36
36–45	40	21
46–55	22	12
56–65	13	7
> 65	6	3
Total	191	100

**FIGURE 1:**
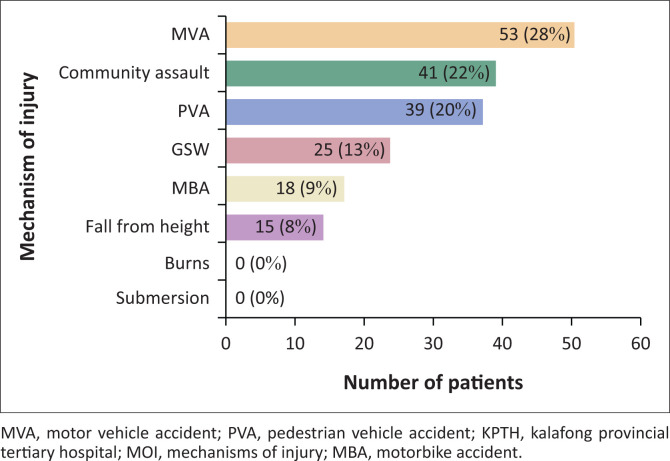
Mechanisms of injury related to frequency of patients presenting to KPTH with severe polytrauma.

Figure 1 demonstrates that the majority of severe polytrauma patients who presented to KPTH during the study period were because of MVAs (28%), mob assault (22%) and PVAs (20%). Gunshot wounds and falls from a height accounted for 9% and 8%, respectively. No severe burns or submersion injuries were found in the study population. Four patients who presented with burns were excluded as their ISS was < 16 and 8 patients with submersion injuries were excluded as they were children (< 18 years). Of the 53 patients involved in MVAs, 24% were ejected from the vehicle.

Of the 191 patients studied, the mean ISS was 39 with a standard deviation of 11. The maximum ISS was 66.

One-way ANOVA was used to compare the mean ISS of the various MOI. There was a statistically significant difference in mean ISS between the various MOI with a *p* = 0.0045. Pedestrian vehicle accidents had the highest mean ISS of 45, followed by MBA (40), mob assault (38) and MVA (37). Gunshot wound (35) and fall from height (35) had the lowest mean ISS.

Table 3 shows that most patients admitted to ICU were patients involved in MVAs (9.95%), PVAs (8.38%) and mob assaults (8.38%), respectively. One patient involved in a PVA was transferred to the ICU at a referral hospital (Steve Biko Academic Hospital).

Of the patients who were taken to theatre straight from the ED, the majority suffered GSWs, MVAs and PVAs, amounting to 6.81%, 6.28% and 3.66%, respectively.

Most patients who fell from a height were admitted to HCU (3.6%), followed by ICU (2.6%). Only one patient was taken to theatre straight from the ED.

Of the 14.14% of patients who demised, 4.71% were involved in mob assaults and 4.19% were because of PVAs. This correlates with the MOI with the highest mean ISS.

**FIGURE 2:**
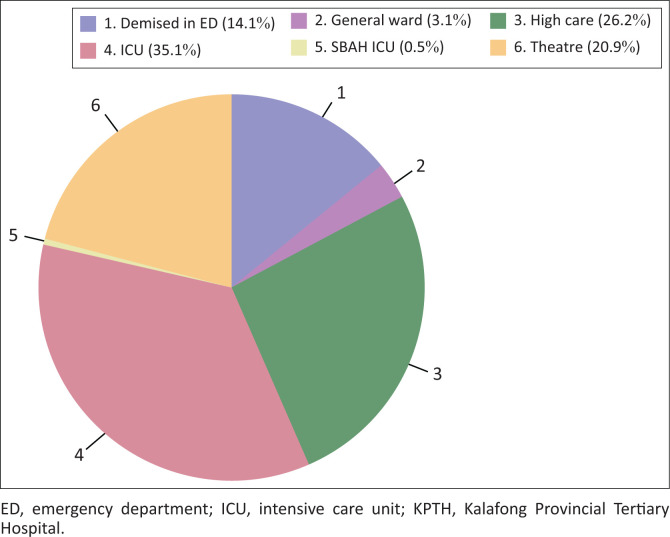
Pie chart illustrating the overall initial disposition of severe polytrauma patients at KPTH.

Figure 2 illustrates the overall initial disposition of severe polytrauma patients at KPTH.

The ED waiting time for admission after the patients have been seen by ED doctors ranged between 1 h and 12 h. While 25% of the patients were admitted within 4 h of arrival to the ED, 50% were admitted within 8 h and 75% of the patients were admitted within 9 h of presentation to the ED (Table 2).

**TABLE 2:** Comparison of the mean Injury Severity Score and mechanisms of injury.

Mechanism	Mean ISS	Standard deviation	Frequency
GSW	35	7.2	25
MBA	40	12.8	18
Mob assault	38	9.8	41
MVA	37	11.6	53
PVA	45	12.4	39
Fall from height	35	9.8	15

**Total**	**39**	**11.2**	**191**

MVA, motor vehicle accident; PVA; pedestrian vehicle accident; ISS, Injury Severity Score; MBA, motorbike accident.

**TABLE 3:** Mechanisms of injury in relation to the initial disposition of severe polytrauma patients.

Mechanism	GW		HCU		ICU		SBAH		Theatre		Patients that demised in ED		Total	
	*n*	%	*n*	%	*n*	%	*n*	%	*n*	%	*n*	%	*n*	%
GSW	0	0.00	6	3.14	4	2.09	0	0.00	13	6.81	2	1.04	25	13.09
MBA	1	0.52	4	2.09	7	3.66	0	0.00	3	1.57	3	1.57	18	9.42
MOB ASS	2	1.04	11	5.76	16	8.38	0	0.00	3	1.57	9	4.71	41	21.47
MVA	2	1.04	16	8.38	19	9.95	0	0.00	12	6.28	4	2.09	53	27.77
PVA	1	0.52	6	3.14	16	8.38	1	0.52	7	3.66	8	4.19	39	20.42
Fall from height	0	0.00	7	3.66	5	2.62	0	0.00	2	1.04	1	0.52	15	7.85

**Total**	**6**	**3.14**	**50**	**26.18**	**67**	**35.08**	**1**	**0.52**	**40**	**20.94**	**27**	**14.14**	**191**	**100.00**

ED, emergency department; MVA, motor vehicle accident; PVA, pedestrian vehicle accident; ICU, intensive care unit; GW, general ward; HCU, high care unit; GSW, gunshot wound; MOB ASS, mob assault; MBA, motorbike accident; SBAH, Steve Biko Academic Hospital.

One-way ANOVA was utilised to assess whether the waiting period to final disposition differed significantly between groups of mechanism of injury. The results presented in Table 4 demonstrate that there is a statistically significant relationship between MOI and waiting period *p* < 0.0001.

**TABLE 4:** Comparison of the mean waiting time to be admitted from Kalafong Provincial Tertiary Hospital emergency department to the final disposition.

Mechanism	Mean (hours)	Standard deviation	Frequency
GSW	4.2	2.5	25
MBA	6.6	2.8	18
Mob assault	7.4	2.5	41
MVA	7.7	2.6	53
PVA	6.4	2.8	39
Fall from height	7.3	2.4	15

**Total**	**6.8**	**2.8**	**191**

MBA, motorbike accident; MVA, motor vehicle accident; PVA, pedestrian vehicle accident; GSW, gunshot wound.

Over half (52%) of patients who sustained GSWs were taken quickly to theatre. Patients who required admission to ICU (mostly MVA, mob assault and PVA) waited between 6.4 h and 7.7 h in the ED for admission.

## Discussion

In this study, we observed the MOI and the disposition of severe polytrauma patients presenting to KPTH ED from 01 July 2018 to 31 December 2019.

Most patients (62%) who presented at KPTH ED with severe polytrauma were males. This is similar to a South African study conducted by Hardcastle et al.^2^ who found that injuries occurred mostly among young healthy men of low socio-economic status. Our results also demonstrated that the majority of patients (36%) were between the ages of 26 years and 35 years. Patients who were older than 65 years of age presented least (3%). These findings are consistent with Alberdi et al.^4^ who found that trauma is one of the leading causes of death in people younger than 40 years of age worldwide.

Saggie^1^ states in the Journal of Trauma, that SA has one of the highest MVA rates in the world. Our study concurred, demonstrating that the majority of patients who presented to KPTH with severe polytrauma were because of MVAs (28%). Mob assault and PVAs were a close second at 22% and 20%, respectively. This is congruent with Hardcastle et al.^2^ who found that injuries in LMICs are largely transport or violence related. The South African Police Service’s crime statistics from 01 April 2019 to 31 March 2020 indicates that at least 1202 people died as a result of mob assault.^9^ A possible explanation for the high number of mob assaults might be a lack of faith in the law-enforcement system by the public. Community members are known to take the law into their own hands by uniting and assaulting those who they deem guilty of crimes.

In our study 24% of the patients involved in MVAs were ejected from the vehicle. This implies that adherence to the law with regard to seatbelt use is poor in our population.

Interestingly, although our study found a relatively low number of patients involved in MBAs (9% in total), this group came second only to PVAs in terms of ICU admissions and mean ISS. Patients involved in MBAs had ICU admission rates of 38.88% and a mean ISS of 40. Patients involved in PVAs had the highest ICU admission rates of 41.03% and the mean highest ISS of 45. Our study found the association between mechanism of injury and severity of injuries to be statistically significant with a *p*-value of 0.0045.

In 2014, Alberdi et al.^4^ investigated the epidemiology of severe trauma and found that major trauma remains a significant cause of morbidity and mortality in developed and developing countries. Our results are in keeping with this finding. In our study, 36% patients were admitted to ICU, 26% to HC and 21% taken to theatre. This demonstrates that these patients had critical injuries warranting high levels of specialist care and intensive nursing. Only 3% of patients were admitted to a general ward. Fourteen per cent of patients demised in the ED while awaiting admission or theatre.

Norman et al.^5^ conducted a study in South Africa using data from an Actuarial Society of South Africa census and from the National Injury Mortality Surveillance System. They demonstrated a high mortality rate in males involved in interpersonal violence and in females involved in road traffic injuries. In our study, the overall mortality rate was 14%. Interpersonal violence in the form of mob assault and GSW accounted for 41% of deaths with 10 of the 11 patients who demised being male (9 mob assault patients and one GSW patient), which concurs with the study conducted by Norman et al.^5^ Road traffic accidents made up 56% of deaths in our study. Thirty per cent of these fatalities were females. Most females who demised were involved in PVAs. The outreach system of the ICU team in our study population, whereby the ICU team assess and treats the patient in the ED while awaiting ICU admission, may have contributed to a lower mortality rate than might otherwise have occurred.

A multicentre German study conducted by Bohmer et al.^10^ found that a high ISS was associated with a prolonged LOS in ICU. Their mean ISS was 21.9 and every additional 5 points added 1 day in the ICU. Our study observed that the mean ISS of patients who were admitted to ICU ranged from 37 years to 45 years. This emphasises the severity of injuries in the South African context, coupled with a higher demand for and longer stays in ICU.

The majority (33%) of the patients who were taken to theatre immediately from the ED sustained GSWs. Of the 25 patients who sustained GSWs, 54% went directly to theatre while 8% demised in the ED. A further 15% of those who sustained GSWs went to ICU and 23% went to HCU. There were no general ward admissions for this group. These findings suggest the acute and severe nature of injuries sustained by GSWs. Despite this, the mean ISS in the GSW group was the lowest in our population, at 35. The underscoring of the ISS in penetrating injury has been suggested in numerous publications and our findings concur.^11,12^ A well-documented limitation of the ISS equation is that the ISS does not consider multiple injuries within the same body region.^12^ Tobin et al.^11^ investigated alternative trauma scores to predict mortality following penetrating GSWs. They demonstrated that for GSWs, the Urban Injury Severity Score (UISS) provided a better correlation for death and LOS in hospital than the ISS or New Injury Severity Score (NISS).^11^ Smith et al.^12^ compared the ISS and the NISS as predictors of hospital mortality in patients with penetrating trauma. They demonstrated that the NISS is a superior scoring system for patients with penetrating injuries.^12^ Furthermore, current literature on trauma scores demonstrates that anatomical trauma scores (ISS and NISS) predict ICU admission better while physiological trauma scores (Revised Trauma Score) predict mortality better.^13^

The timeframe prescribed by the Society of Critical Care Medicine (SCCM) for admission to the ICU from the time of decision to admit, made in the ED, is 6 h.^14^ Our study demonstrated that patients waited on average 7.7 h for ICU admission. The delay of ICU admission for ED patients is common in LMIC because of limited ICU resources, overcrowded EDs with a high burden of disease, and access blocked to ICU beds.^15^ A 2022 Greek meta-analysis by Kiekkas et al.^16^ suggests that there is a significant increase in the mortality of critically ill medical and surgical patients with delayed ICU admission. Interestingly, however, a South African retrospective study in 2021 observing critically ill medical and trauma patients, demonstrated that a delay to ICU admission from the ED was not associated with increased mortality.^14^ This study population included > 70% surgical patients of which > 40% were trauma related.^14^ This finding is supported by another LMIC investigating the impact of delayed ICU admission in patients presenting to a level 1 trauma centre in India.^15^ It appears that both patient and institutional factors may be attributable to this discrepancy between mortality with delayed ICU admissions in high income and LMIC. The authors speculate that one of the reasons for this discrepancy could be that surgical or trauma-related injuries often occur in younger, healthy patients.^14^ It seems that the underlying medical condition of the patient has a greater impact on mortality than the LOS in the ED before ICU admission.^14^

Our study demonstrated an impressive overall ED LOS of severe polytrauma patients ranging between 1 h and 12 h. Twenty-five per cent of patients were admitted within 4 h of arrival to the ED, 50% within 8 h, 75% within 9 h and 100% by 12 h. A retrospective Qatari study^17^ published in the World Journal of Emergency Medicine 2023 showed that 23% of trauma patients were admitted from the ED within 4 h, 74% within 12 h, 91% within 24 h and 9% stayed in the ED for more than 24 h. Even though the study population is not identical (trauma patients in Qatar versus severe polytrauma patients in our study), our results are applaudable when compared to the results of this high-income country.

### Limitations

- The process of data collection was manual. The records were archived by administrative clerks and 30 files per day were collected from data stores by the researcher. Although performed as comprehensively as possible, four files from the register were untraceable.
- Whether the patient was ejected from the vehicle or not was not always stipulated in the notes of every patient involved in an MVA. This number may be underestimated.
- As only the initial disposition of patients from the ED was recorded, if a patient was taken to theatre after being admitted to a general ward, ICU or HCU, it was not recorded as a theatre disposition.

### Recommendations

- The study recommends that the hospital develops a trauma surgical team that is on call daily. This will alleviate the workload of the general surgeon on call and limit delays in seeing trauma patients.
- The authors recommend that electronic recordkeeping be employed by KPTH.
- The file archiving and retrieval method at KPTH should be finessed so that 100% data collection is made possible for any retrospective study such as this.

## Conclusion

Most patients who presented at KPTH with severe polytrauma were males. The predominant age group was 26–35 years of age. Motor vehicle accidents were found to be the most prevalent MOI. Patients involved in PVAs were most severely injured according to the ISS.

Most patients were admitted to ICU (36%) and HCU (26%). Fourteen per cent of patients demised in the ED before admission and most of these were as a result of head injuries from community assaults.

The waiting time for ICU admissions ranged between 6.4 h and 7.7 h in our unit. All severe polytrauma patients were admitted by 12 h.

Statistically significant associations were found between MOI and ISS (*p* = 0.0045), and between MOI and disposition (*p* = 0.0001).

## Appendix 1

**TABLE 1-A1:** Injury Severity Score.

Head and neck worst injury	Face worst injury	Chest worst injury	Abdomen worst injury	Extremity (including pelvis) worst injury	External worst injury
Minor (+1)	Minor (+1)	Minor (+1)	Minor (+1)	Minor (+1)	Minor (+1)
Moderate (+2)	Moderate (+2)	Moderate (+2)	Moderate (+2)	Moderate (+2)	Moderate (+2)
Serious (+3)	Serious (+3)	Serious (+3)	Serious (+3)	Serious (+3)	Serious (+3)
Severe (+4)	Severe (+4)	Severe (+4)	Severe (+4)	Severe (+4)	Severe (+4)
Critical (+5)	Critical (+5)	Critical (+5)	Critical (+5)	Critical (+5)	Critical (+5)
Unsurvivable (+6)	Unsurvivable (+6)	Unsurvivable (+6)	Unsurvivable (+6)	Unsurvivable (+6)	Unsurvivable (+6)

*Source:* Baker S. Injury Severity Score (ISS) [homepage on the Internet]. [cited n.d.]. MDCALC. Available from: https://www.mdcalc.com/calc/1239/injury-severity-score-iss

## Appendix 2

**TABLE 1-A2:** Abbreviated Injury Scale (AIS)

AIS code	Description
1	Minor
2	Moderate
3	Serious (non-life-threatening)
4	Severe (life threatening, survival probable)
5	Critical (survival uncertain)
6	Unsurvivable (with current treatment)

*Source:* Baker S. Injury Severity Score (ISS) [homepage on the Internet]. [cited n.d.]. MDCALC. Available from: https://www.mdcalc.com/calc/1239/injury-severity-score-iss
